# Health Care Reform Now! A Prescription for Change

**Published:** 2008-09-15

**Authors:** Michael Givel

**Affiliations:** University of Oklahoma, Norman, Oklahoma

**Figure:**
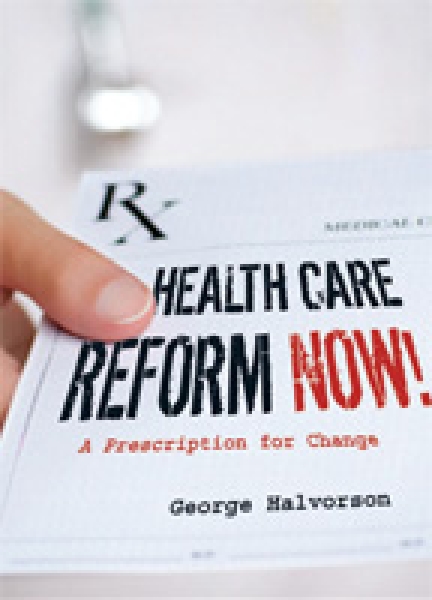


In *Health Care Reform Now!*, George Halvorson, who is the chair and chief executive officer (CEO) of the Kaiser Foundation Health Plan and Kaiser Foundation Hospitals Inc in Oakland, California, and CEO of HealthPartners in Minneapolis, calls for 2 major reforms of the US health care system: universal health care coverage for all citizens, including 46 million Americans who do not have health insurance, and cost-savings approaches for the private health insurance industry, which would play a crucial role in providing health services.

Halvorson proposes a legal mandate that would require all citizens to obtain health coverage. This coverage would be acquired through individual purchase of health insurance, employer provision of health coverage, Medicare, Medicaid, and a price-regulated entitlement program (for the working poor and parts of the middle class) that is based on income and covered by the government or employers for those not covered by Medicaid or Medicare. Other features of Halvorson's universal health care proposal are a small penalty for employers that do not insure their employees and the use of tax records to verify that people are insured.

Halvorson also calls for market incentives to reform administrative practices of the health insurance industry in the form of enhanced and cost-saving systems that allow for more effective data sharing among health care providers. He believes cost can be contained by more effective evidence-based measurements of health care and by community-oriented preventive medicine approaches, such as obesity control, to prevent health problems early. Halvorson further urges the health insurance industry to promote a buyer-focused approach based on effective competition for health services. He recommends charging all patients a large, flat deductible, penalizing them financially for risky behavior and rewarding them for healthy behavior, and prepaying health providers to perform health services at a fixed cost. Under Halvorson's proposal, additional costs to the health insurance industry would be paid for by a health sales tax applied only to health services.

Halvorson's proposal for universal health care is fairly congruent with the universal health care system enacted in 2006 in Massachusetts. The Massachusetts model mandates health care coverage for all Massachusetts citizens; financial penalties for businesses, except very small ones, that fail to cover their employees; subsidized, but not free, health care for people who earn less than 3 times the poverty level, excluding Medicaid recipients; financial penalties for citizens who do not purchase health care; and the promotion of health savings accounts (1).

However, just 1 year after the passage of the Massachusetts law, as many as 100,000 to 300,000 previously uninsured Massachusetts citizens were not enrolled in the program (2). The Massachusetts health program also does not address excessive administrative costs because of the myriad health care providers. The Massachusetts approach restricts patients' choice of doctors because fewer doctors are available to those who are enrolled in low-cost plans. Finally, private insurers in Massachusetts plan on raising their premium rates 8% to 13% in 2008, which is approximately twice the 2007 national average (2). This increase raises the issue of what effect the Massachusetts health program and Halvorson's proposal would have on the health insurance industry's willingness to set reasonable premiums, deductibles, and co-pays, particularly for the working poor and the middle class.

Curiously, or perhaps not, Halvorson's book, in its long discussion on how to create a more efficient and economical health insurance industry, emphasizes that the rise of health care costs is due to buyer demand and lack of data efficiency but ignores the role of health care providers in the rise of costs. Specifically, Halvorson's proposal fails to acknowledge that administrative costs represent 31% of the annual amount spent per capita on health care in the United States (3), a portion of which is attributable to administrative overlap among the large number of private health care providers. His proposal does not address the increasing cost of health insurance in an environment of high industry profits — Americans spend more per capita on health care than do citizens of most other industrialized nations (4) — or the current trend of shifting costs from the health insurance industry to patients in the form of increasingly higher premiums, deductibles, and co-pays, as is occurring in Massachusetts (5). Halvorson's proposal for a health sales tax does not mention that this tax is regressive and falls disproportionately on low- and moderate-income people. Also unaddressed is the role of medical malpractice lawsuits in rising health care costs.

In short, this book raises many crucial questions that it does not answer. In its failure to examine all the major factors related to health care costs and universal access, it represents a decidedly one-sided and unbalanced view of the issues facing the US health care system. Consequently, it should be read from the perspective of a partisan proposal that — probably not coincidentally — does not unduly affect the current private health care market and industry.
